# A comprehensive approach to studying motor planning and execution using 3D-printed objects and motion tracking technology

**DOI:** 10.3389/fnhum.2025.1620526

**Published:** 2025-06-25

**Authors:** Alexander Vyazmin, Sangram Behera, Geok Lan See, Victoria Moiseeva, Matteo Feurra

**Affiliations:** ^1^Centre for Cognition and Decision Making, Institute for Cognitive Neuroscience, HSE University, Russian Federation, Moscow, Russia; ^2^Cajal Neuroscience Centre (CNC), Consejo Superior de Investigaciones Cientificas (CSIC), Madrid, Spain; ^3^Unit Psikologi Klinikal, Hospital Rehabilitasi Cheras, Kuala Lumpur, Malaysia

**Keywords:** motor planning, grasping kinematics, anticipatory control, movement segmentation, motion tracking, 3D-printed objects, neurorehabilitation

## Abstract

**Background:**

Motor planning critically supports efficient hand grasping and object manipulation, involving the precise integration of sensory cues and anticipatory motor commands. Current methods often inadequately separate motor planning from movement execution, thus limiting our understanding of anticipatory motor control mechanisms.

**Objective:**

This study aimed to establish and validate a structured methodological approach to investigate motor planning and execution during grasping tasks, using advanced motion tracking technology and standardized 3D-printed geometric objects.

**Methods:**

Twenty-one participants performed a grasp-and-place task, requiring manipulation of abstract, non-semantic objects under varying rotation angles (0°, 90°, 180°, 270°). High-resolution kinematic data were captured using an infrared motion tracking system (Smart-DX, BTS Bioengineering, Italy). Novel computational analyses segmented each trial into distinct phases: total movement, movement initiation, reaching, maximal grasp aperture, and object placement. Wrist path length and execution time of each phase were statistically analyzed to assess the influence of object rotation on motor planning and execution.

**Results:**

Object rotation significantly impacted motor planning, as evidenced by prolonged initiation times and altered grasp-related temporal parameters. Specifically, movements involving rotation demonstrated increased movement initiation times, greater grasp apertures, extended placement durations, and longer wrist trajectories compared to non-rotated conditions. Interestingly, symmetrical rotations (180°) facilitated faster and more efficient movements compared to asymmetrical rotations (90°, 270°).

**Conclusion:**

Our validated methodological framework enables precise isolation and assessment of motor planning processes during grasping movements. This paradigm provides robust tools for fundamental motor control research and has potential clinical applications for evaluating motor planning deficits in patients with neurological impairments.

## Introduction

Hand grasping and object manipulation are fundamental motor actions essential for interacting with the environment, communicating, and performing daily activities such as eating and dressing (MacKenzie and Iberall, 1994). These activities integrate sensory input, motor planning, and execution processes. Investigating these mechanisms provides critical insights into how the nervous system coordinates complex motor behaviors. Motor planning is particularly crucial, as it prepares the motor system in advance on the basis of sensory input and task demands, thus shaping movement execution (Jeannerod, 1997; Wong et al., 2015). According to Wong et al. (2015), motor planning involves multiple stages: abstract kinematics, effector trajectory selection, and movement specification. These stages collectively formulate a set of motor commands for execution, especially relevant when multiple potential movement strategies exist for achieving a goal. The influence of motor planning on reaction time, grasp aperture, and hand trajectory is significant, particularly as task complexity increases (Santello et al., 1998; Castiello, 2005). Impairments in motor planning and grasping are frequently observed in neurological disorders. For example, patients with Parkinson's disease exhibit reduced movement intensity and coordination deficits that significantly affect grasping, tool use, and fine motor skills (Fasano et al., 2022; Vissani et al., 2021). Stroke patients often experience weakened grip strength, delayed movement initiation, and altered grasp aperture (Parry et al., 2019), whereas peripheral neuropathies induced by treatments such as chemotherapy disrupt hand coordination, significantly impacting daily function (Osumi et al., 2019). Considering that up to 60% of stroke survivors suffer persistent motor deficits (Nowak, 2008), enhancing our understanding and rehabilitation of grasping impairments is crucial for improving patient outcomes and quality of life.

Various methodologies have been employed to study motor planning in grasping tasks, including reaction time paradigms, electrophysiological recordings, and neuroimaging techniques. Reaction time studies measure the delay between stimulus presentation and movement initiation, providing indirect evidence of planning processes (Delmas et al., 2018). However, these approaches often lack the spatial and temporal resolution necessary to support detailed temporal segmentation of movement phases based on positional data. Electrophysiological techniques such as electroencephalography (EEG) and transcranial magnetic stimulation (TMS) provide insights into neural activation during motor planning (Zaepffel et al., 2013; Verstraelen et al., 2021), but their ability to track detailed movement execution is limited. Functional neuroimaging, including functional Magnetic Resonance Imaging (fMRI), has been used to identify brain regions involved in motor planning (Hanakawa et al., 2008). However, it lacks the temporal resolution required to analyze movement dynamics in real time.

To address these gaps, motion tracking systems have emerged as essential tools for extracting precise positional data and segmenting movement phases (Betti et al., 2018). Although some studies have employed motion capture systems to analyze reach-to-grasp movements (Verhagen et al., 2012), many protocols do not adequately isolate the motor planning component from execution. Furthermore, few studies have incorporated object manipulation constraints such as rotation, which significantly affect anticipatory control. Research on mental rotation indicates that even imagined object transformations engage motor planning mechanisms, emphasizing the importance of evaluating grasping tasks that require actual physical object rotation (Shepard and Metzler, 1971; Wohlschläger, 2001; Wexler et al., 1998).

Typically, grasping actions performed with everyday objects are influenced by top-down cognitive processes such as object affordances, familiarity, and semantic meaning, which can significantly affect motor performance (Rosenbaum et al., 2007). To minimize these cognitive influences and better isolate the motor planning and execution processes, we deliberately used abstract, non-sensical geometric objects devoid of semantic content. This methodological decision allowed us to precisely control and examine motor processes independently of top-down cognitive biases or familiarity effects.

To address these limitations, we introduce a new methodological framework for isolating and examining motor planning processes in grasping. The task was designed to manipulate anticipatory control demands through object orientation, allowing us to dissociate simpler from more complex actions in a controlled and reproducible manner. By analyzing the movement initiation time, reaching time, maximal grasp aperture, object movement time, total movement time, and wrist path across different rotation conditions, we distinguish between simpler (non-rotated) and more complex (reoriented) movements. This distinction helps isolate motor planning components from execution (Paulun et al., 2016). Our approach relies on high-resolution motion tracking and aims to provide a structured basis for analyzing distinct phases of grasping behavior. This study presents our methodological rationale and validation. We aim to establish a robust and replicable protocol for investigating motor planning in grasping, providing a foundation for future research and clinical integration.

This study presents our methodological framework, including task design, data acquisition, and analysis protocols. We provide validation through temporally segmented positional data derived from motion tracking, demonstrating how object orientation influences movement phase durations and motor planning demands. Our goal is to establish a robust and replicable approach that can be widely adopted for studying the temporal structure of grasping movements, based on positional tracking data, in both research and clinical contexts. This work builds upon and extends our previously published work (Vyazmin et al., 2024). In the present study, we replaced the original objects with precisely designed 3D-printed versions to ensure consistency in shape, weight, and texture, thus minimizing potential variability in grasping dynamics. Furthermore, we implemented a new analytical approach that segments grasping movements into distinct temporal phases based on positional tracking data, enabling a more detailed evaluation of motor planning processes.

## Methods

### Participants

Twenty-one participants were included (13 females), with a mean age of 24.23 ± 3.49 years. All participants, except for one, were right-handed and had no history of neurological, psychiatric, or other chronic illnesses. They also had no right-hand injuries and were not taking medication for chronic conditions. Additionally, all participants had either normal or corrected-to-normal vision and no diagnosed eye diseases. They were instructed to refrain from consuming alcohol for 24 h prior to the experiment. All participants signed informed consent to participate in the study and received monetary compensation. The study was performed in accordance with the recommendations of the Declaration of Helsinki. The final sample size (*N* = 21) was determined based on effect sizes from our previous study (Vyazmin et al., 2024), which reported large effects (η^2^ ranging from 0.25 to 0.80) for key movement parameters. Similar sample sizes were also employed in comparable studies (Betti et al., 2018; Paulun et al., 2016), supporting the adequacy of our design.

### Task

The primary objective of this task was to examine the different stages of hand grasping movements while performing actions involving object placement with varying rotational requirements. Specifically, some actions required the participant to rotate the object before placement, whereas others did not. To achieve this goal, a specialized experimental task incorporating multiple rotation conditions was developed.

The participants were instructed to grasp specially designed objects with non-sensical geometric shapes and place them onto a corresponding cardboard plate. During this process, the object had to be rotated to match one of four predefined angles: 0°, 90°, 180°, or 270°.

**Figure 1:**
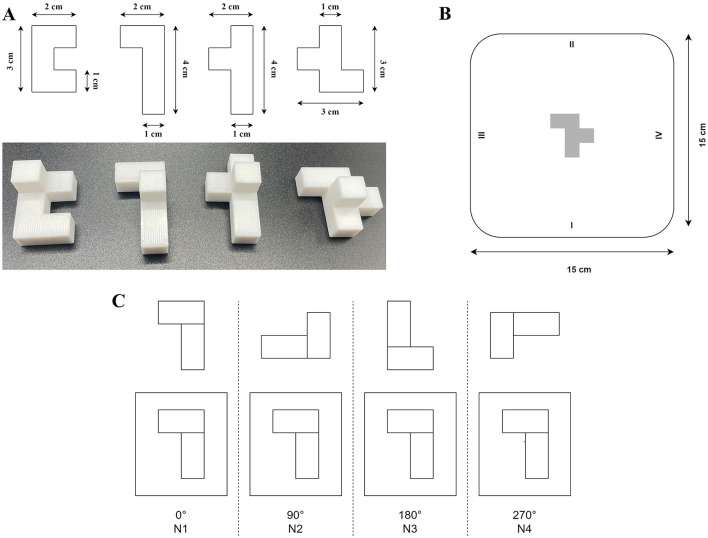
**(A)** Four distinct 3D-printed objects featuring abstract, non-sensical geometric shapes. All objects were designed with identical surface areas and volumes to minimize variability during grasping tasks. **(B)** Example of a rectangular cardboard plate used during the experimental task. The plate features an image of one of the objects centrally positioned, with numerical markers at its edges (1–4) indicating rotation angles to guide precise orientation according to trial requirements. **(C)** Illustration of experimental conditions categorized by object rotation angles. The top row depicts the initial orientation of an example object at the trial's start, while the bottom row shows the corresponding orientation required for object placement. Conditions are labeled according to rotation angles: 0° (N1), 90° (N2), 180° (N3), and 270° (N4). Adapted from Vyazmin et al. (2024), Neuromuscular Diseases, with permission.

For this task, we developed four distinct 3D-printed objects with non-sensical geometric shapes and four rectangular cardboard plates (15 × 15 cm) featuring images of one of these objects at the center (Figure 1). All the objects were designed to have the same volume to eliminate size-related variability. All objects were composed of five identical cubes (1 cm3 each), resulting in a total volume of 5 cm3 per object. The cubes were arranged in a single plane but in varying configurations. An additional identical cube was placed centrally on top of each object for tracker placement, without altering the graspable structure. Before each trial, one object and its corresponding cardboard plate were placed on the experimental table while the participant's view was blocked to prevent prior knowledge of the setup. During the task, all four objects were presented in a randomized order. Each object was rotated to one of the four angles (0°, 90°, 180°, or 270°), and the corresponding cardboard plate was also randomly rotated to one of these angles. Thus, for each trial, the object and its corresponding image on the plate could be rotated relative to each other by 0°, 90°, 180°, or 270° (Figure 1). This setup resulted in multiple combinations of object and plate orientations across trials.

The participants were required to:

1. Grasp the object using their thumb and index finger.
2. Rotate the object (if needed) to match the orientation of the image on the plate.
3. Place the object onto the plate, aligning it with the image's orientation.

To control for potential learning effects, a total of 64 randomized trials were conducted, covering various combinations of objects and rotation angles.

### Experimental setup

**Figure 2:**
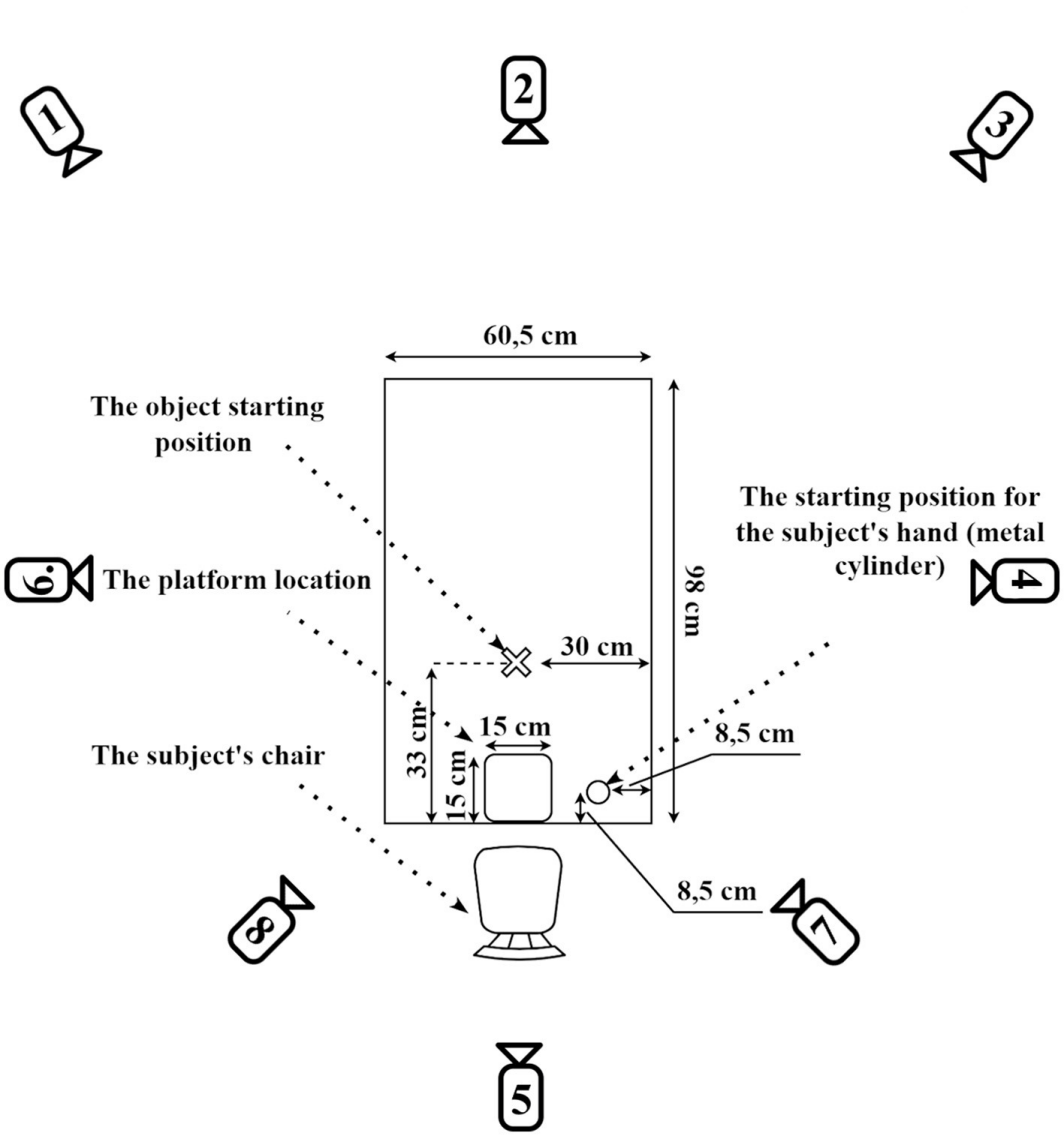
Schematic diagram of the experimental setup with camera positions. The experimental table measures 90 cm by 60.5 cm. The participant's hand starting position is marked 5.5 cm from the shorter edge and 8.5 cm from the longer edge of the table. The target plate (15 cm × 15 cm) is positioned centrally along the short edge, with its bottom edge aligned precisely with the table edge. The initial position for the experimental object is located 33 cm from the short edge and 30 cm from the long edge, ensuring consistent placement across trials. Numbered circles indicate the placement of eight infrared cameras used for motion tracking. Cameras 1, 3, 7, and 8 were positioned at a height of 1.5 m, while cameras 2, 4, 5, and 6 were placed at a height of 2 m. This configuration ensured comprehensive three-dimensional tracking coverage during task execution. Adapted from Vyazmin et al. (2024) Neuromuscular Diseases, with permission.

The participants were comfortably seated near an experimental table (98 × 60.5 cm). A metal cylinder (2 cm height, 1 cm diameter) was fixed on the table surface, marking the starting position for the participant's right hand. The table also had predefined locations for placing the experimental object and the corresponding cardboard plate (Figure 2).

**Figure 3:**
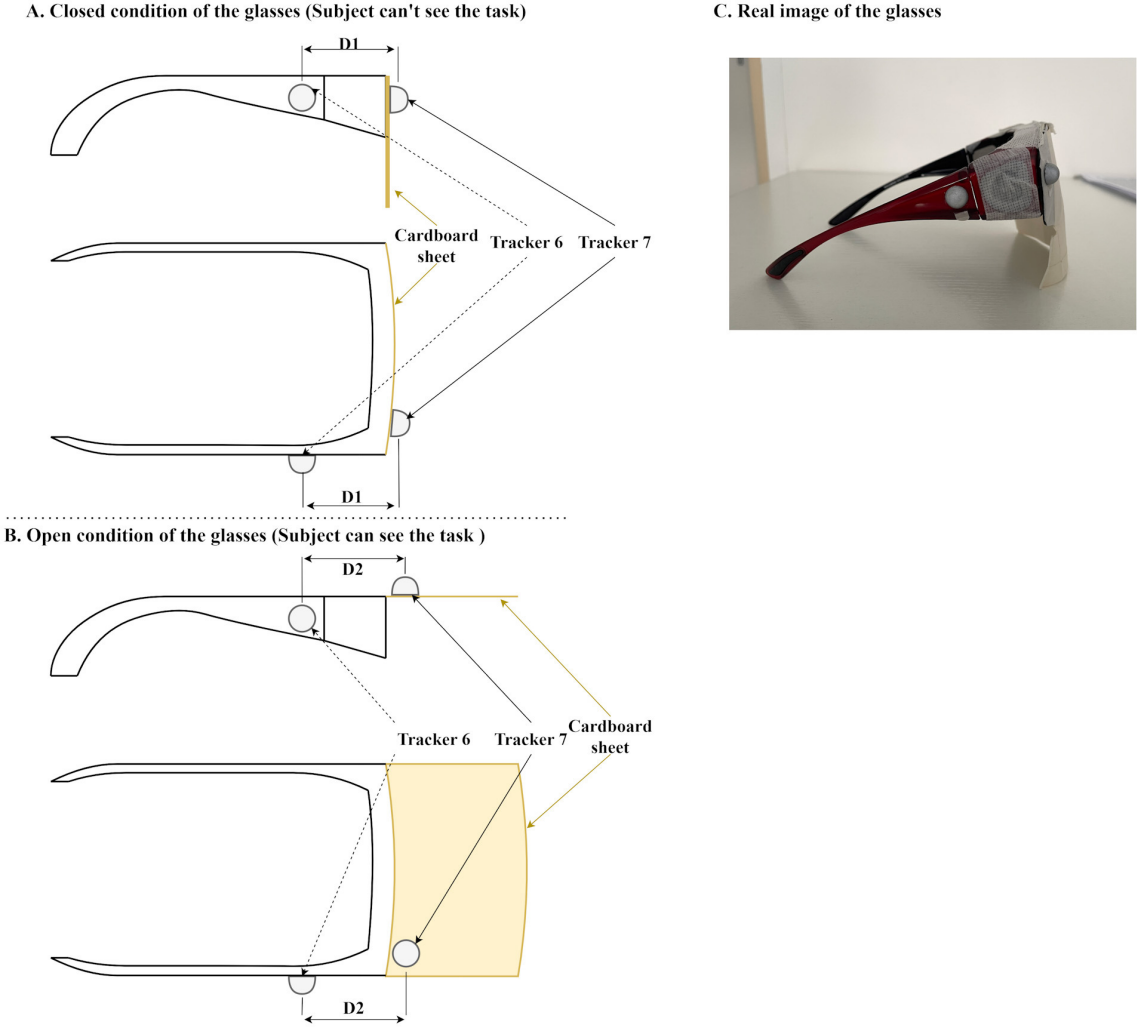
Specialized glasses used during the experiment. **(A)** Schematic illustration of the glasses in the closed condition, obstructing the participant's view of the experimental setup. **(B)** Schematic illustration of the glasses in the open condition, restoring visibility and signaling the start of the trial. In these panels **(A, B)**, D1 indicates the distance between Tracker 7 and Tracker 6 in the closed state, and D2 indicates the increased distance in the open state. The onset of the trial is precisely defined as the moment when the distance between the trackers reaches its maximum (D2). **(C)** Real photographic representation of the glasses.

To ensure that the participants were blind to the experimental setup during the preparation phase, each participant wore specialized glasses with movable lenses (Figure 3). The lenses were covered entirely with cardboard, completely obstructing vision when closed. During the preparation phase, the experimenter, positioned behind the participant, closed these lenses to block the participant's view. Infrared reflective markers were attached to the glasses, enabling precise identification of trial onset based on positional tracking data. To control participants' visual access to the task, occlusion glasses were manually opened by the experimenter. Importantly, the onset of each trial was not defined by the manual gesture but by the maximum distance between two reflective markers attached to the glasses (described in the Motion tracking system section, Tracker 6 and Tracker 7), ensuring precise and reproducible timing across trials. Before each trial, the experimenter arranged the object and cardboard plate according to a predefined randomized trial sequence (Appendix A). This sequence specified:

- The object to be grasped.
- The initial orientation of the object.
- The orientation of the cardboard plate.

The trial sequence was randomized uniquely for each participant to prevent potential order effects.

To guarantee effective randomization, we employed a systematic randomization procedure using Microsoft Excel. All possible stimulus-condition combinations were enumerated, each assigned a random numeric value generated by Excel's built-in RAND() function, producing uniformly distributed random numbers between 0 and 1. Subsequently, the stimuli were sorted based on these random values, ensuring a balanced yet randomized sequence for each participant.

### Trial procedure

Each trial consisted of four sequential phases:

Preparation phase: While the participant's lenses remained closed, the experimenter placed the object and cardboard plate according to the randomized trial sequence.

#### Trial initiation

The experimenter opened the participants' lenses, signaling that the participants would start the trial.

#### Action phase

Participants performed the following steps sequentially:

- The objects were grasped using their thumb and index finger.
- The object was rotated, if necessary, and aligned it with the orientation displayed on the cardboard plate.
- The object was placed onto the corresponding plate.
- The right wrist was returned to the initial starting position.

#### Trial conclusion

**Figure 4:**
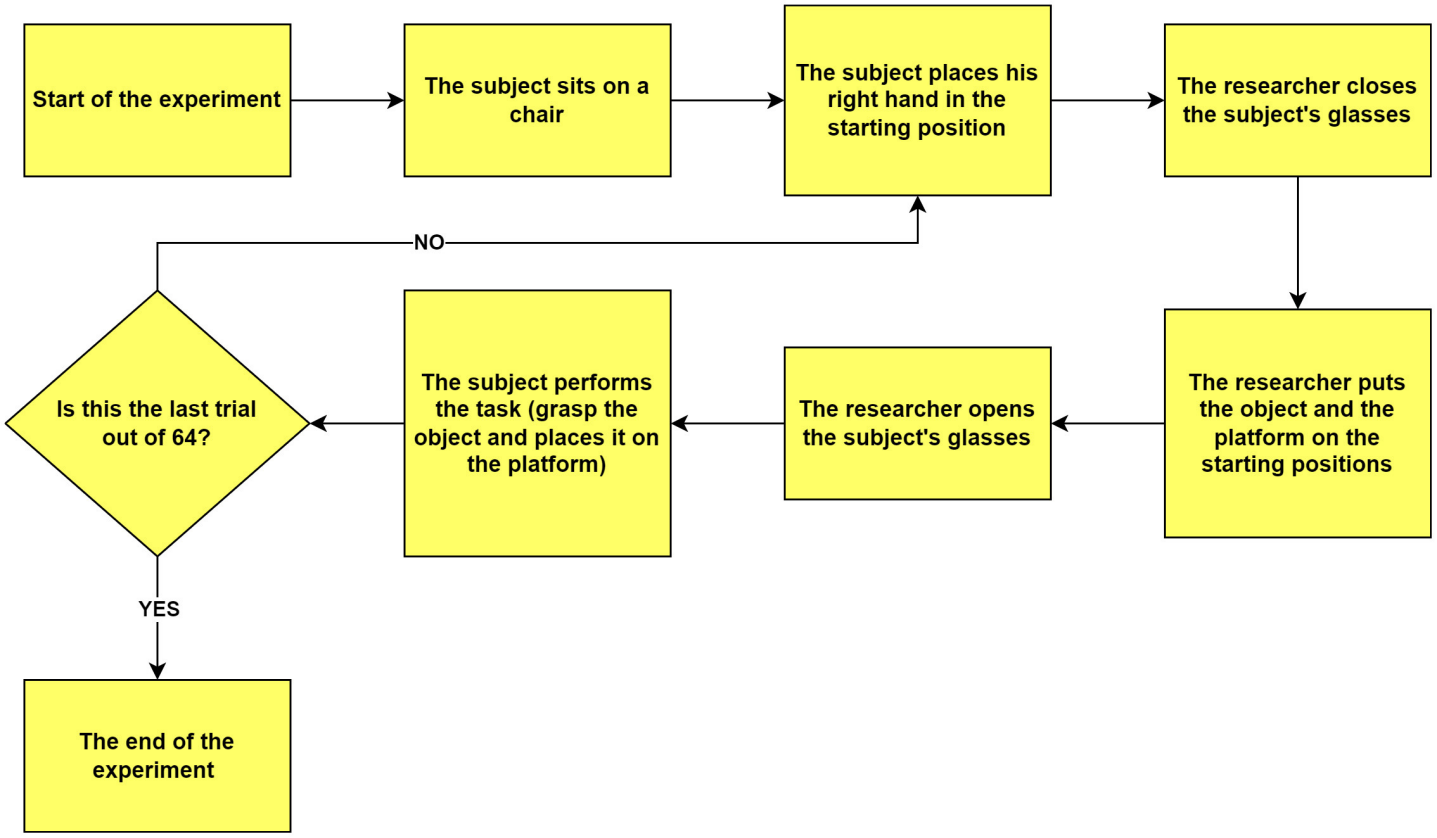
Flowchart illustrating the experimental procedure. The diagram outlines the sequential steps from the beginning to the end of the experiment. Each trial starts with the participant seated, their hand positioned at the designated starting point, and their view obstructed by closed glasses. The experimenter arranges the object and platform in predetermined positions, then opens the participant's glasses to signal trial onset. The participant then performs the grasp-and-place task. This cycle repeats for 64 trials, after which the experiment concludes.

The experimenter closed the lenses, cleared the table, and arranged the setup for the subsequent trial. This procedure was repeated systematically for all 64 trials, as illustrated in Figure 4, ensuring consistency and precision and minimizing experimental bias.

### Motion tracking system

Hand movements were recorded using the Smart-DX motion tracking system (BTS Bioengineering, Italy). This system, consisting of eight infrared cameras, was positioned around the experimental area, as depicted in Figure 2, and recorded at 250 Hz.

To reliably capture 3D coordinates, each infrared marker (tracker) required visibility from at least three cameras simultaneously. Cameras No2, No4, No5, and No6 were positioned at a height of 2 m above the laboratory floor, at a distance of 1.5 m from the center of the experimental table along the perimeter of a rectangle surrounding the table, centered on each side. Cameras No1 and No3 were positioned at a height of 1.5 m above the laboratory floor, at a distance of 2 m from the center of the experimental table, and placed at the corners of a rectangular perimeter around the table. Cameras No7 and No8 were specifically positioned closer to the participants to reduce marker occlusion during subtle wrist movements and were placed at a height of 1.5 m and a distance of 1.5 m from the center of the experimental table.

We used 10 infrared trackers, fixed to anatomical landmarks and experimental objects with double-sided adhesive tape at the following positions:

- Tracker 1: Thumbnail center (right hand).
- Tracker 2: Index fingernail center (right hand).
- Tracker 3: Styloid process of the radius (right wrist).
- Tracker 4: Styloid process of the ulna (right wrist).
- Trackers 5, 8, 9, and 10: On top of each experimental object, ~1 cm above its surface (one tracker per object).
- Tracker 6: Right side of glasses frame.
- Tracker 7: Right side of the glasses' movable lens cover.

**Figure 5:**
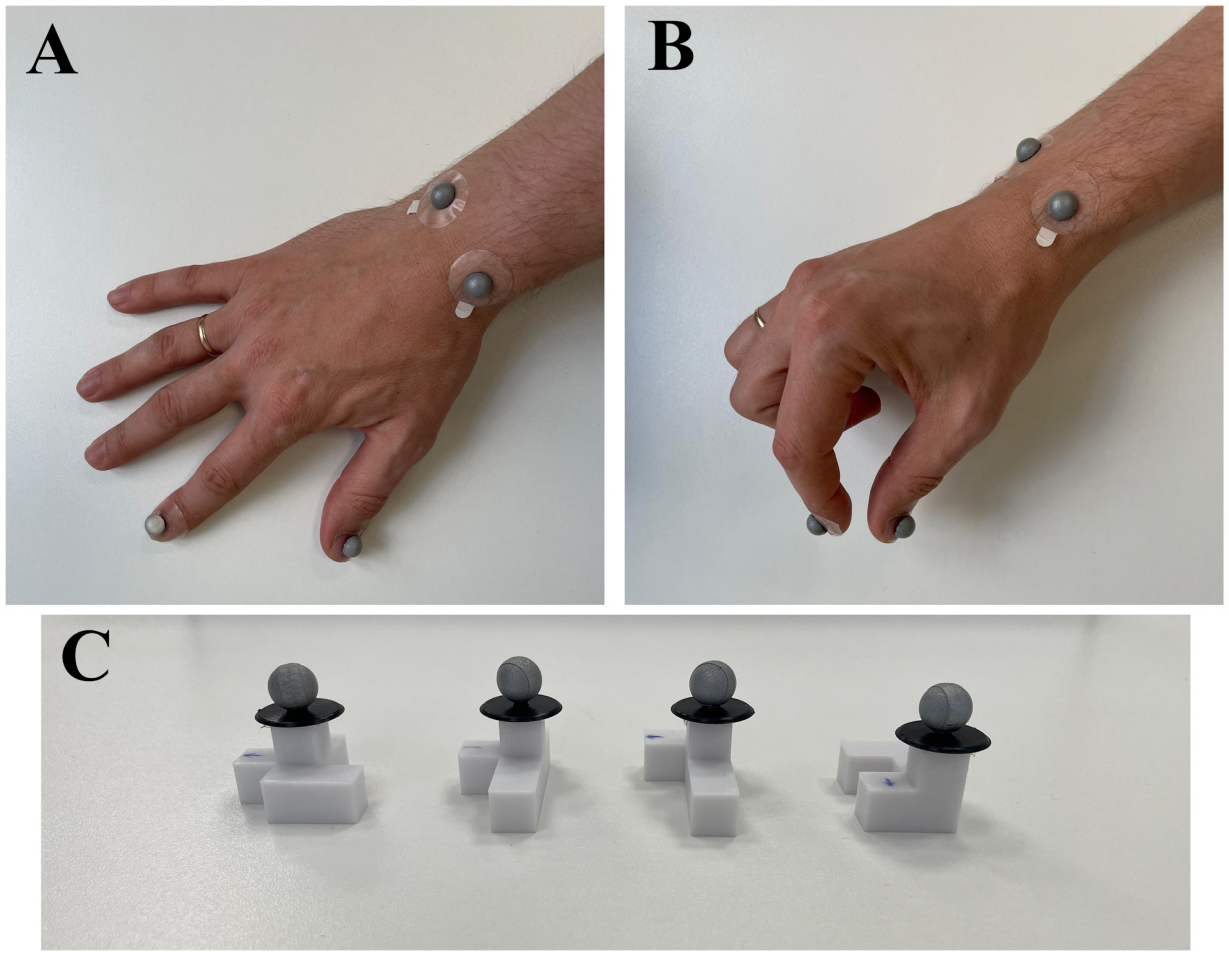
Placement of infrared motion tracking markers on the hand and experimental objects. **(A)** Dorsal view of the right hand showing markers attached to anatomical landmarks: the thumbnail (Tracker 1), index fingernail (Tracker 2), and the styloid processes of the radius and ulna (Trackers 3 and 4). **(B)** Lateral view of the right hand in a prehension posture, illustrating the visibility and alignment of the markers during movement. **(C)** Experimental objects with reflective markers (Trackers 5, 8, 9, and 10) mounted ~1 cm above the surface to ensure clear visibility and accurate tracking during manipulation.

Figure 5 provides a visual reference for the spatial configuration of the tracking system, illustrating hand placement and the positions of the markers on the experimental objects. Trackers 1 and 2 specifically quantified the grasping component, whereas trackers 3 and 4 measured the reaching and placement components. Object-mounted trackers (5, 8, 9, and 10) facilitated indirect measurement of the object's placement phase. Although each object had a unique tracker, it was collectively analyzed as Tracker 5 because the motion tracking system does not distinguish individual markers separately, and only one object was presented per trial.

Glass-mounted trackers (6 and 7) allowed precise detection of trial initiation, marked by the opening of the glasses. The distance between these trackers was shortest when glasses were closed and increased distinctly upon opening, providing a precise timestamp for trial onset.

### Data analysis

The dataset comprised 64 trials per participant. Custom analysis scripts were developed in MATLAB (MathWorks, USA) for detailed trial-by-trial analysis. The raw 3D marker data were smoothed using a Gaussian-weighted moving average filter implemented via the standard MATLAB function “smoothdata” (method: “gaussian,” window length: 30 samples). This approach effectively reduced high-frequency noise while preserving the fidelity of the underlying movement trajectory. Based on the filtered positional and velocity data, we then objectively identified key movement events (reference points) that defined the temporal structure of each trial:

- Glasses Opening Time (T1): Defined as the first point after the maximum distance between Tracker 6 and Tracker 7, when their relative velocity becomes positive, indicating lens opening.
- Wrist Lifting Time (T2): Identified as the initial time after T1 at which the wrist vertical velocity exceeded 0.05 cm/s and remained above this threshold for at least 20 consecutive frames. The wrist position was calculated as the virtual midpoint between Trackers 3 and 4.
- Maximum Grasp Aperture Time (T3): Defined as the second significant aperture peak between the thumb and index finger markers (Trackers 1 and 2) occurring after T1. This approach accounts for two distinct finger-opening cycles consistently observed: initial release from the starting cylinder and subsequent aperture preparation for object grasping, a novel phenomenon requiring further exploration.
- Object Lifting Time (T4): Marked the grasp completion and object placement initiation phase, identified as the first instance post-T3 when the object vertical velocity surpassed 0.01 cm/s for at least 20 frames.
- Object Placement Time (T5): Identified when the vertical velocity of the object first fell below 0.01 cm/s and averaged less than 0.05 cm/s for the next 75 frames, indicating stable placement.

We subsequently calculate the following parameters:

- Movement Initiation Time (T2–T1): Reflecting cognitive decision-making and preparatory motor planning.
- Time to Maximum Grasp Aperture (T3–T2): This represents the complexity of grasp preparation.
- Reaching Time (T4–T2): Measuring the reaching phase duration.
- Object Placement Time (T5–T4): Representing the object placement efficiency.
- Total Movement Time (T5–T1): Including decision-making, motor planning, grasping, and placement phases.
- Wrist Path Length (T2–T4): Reflecting reaching efficiency, computed from the midpoint between the thumb and index finger trackers (1 and 2).

**Figure 6:**
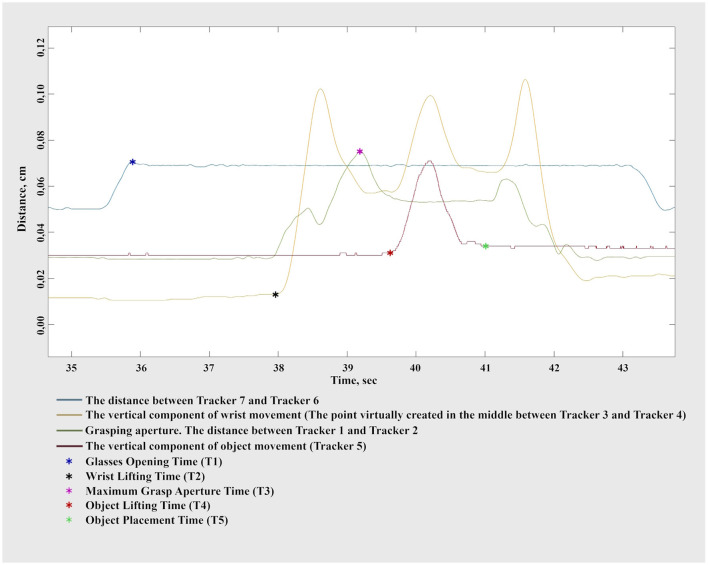
Representative kinematic traces illustrating key events during a single grasp-and-place trial. Distinct lines represent: the distance between Trackers 7 and 6 (glasses), wrist vertical displacement (midpoint between Trackers 3 and 4), grasping aperture (distance between thumb and index finger markers, Trackers 1 and 2), and object vertical displacement (Tracker 5). Colored markers indicate specific temporal events: glasses opening time (T1, blue), wrist lifting time (T2, black), maximum grasp aperture time (T3, red), object lifting time (T4, purple), and object placement time (T5, green).

We show the dynamics of a single trial movement along with the corresponding kinematic graphs and events in Figure 6. To investigate the effects of different object rotation angles, trials were grouped into four conditions based on rotation requirements: N1 = 0° (no rotation), N2 = 90°, N3 = 180°, and N4 = 270°. In conditions N2–N4, participants had to rotate the object to align it with the target orientation on the placement board (see Figure 1). One-way repeated-measures ANOVAs were conducted separately for each parameter, specifically total movement time (N1, N2, N3, N4), movement initiation time (N1, N2, N3, N4), reaching time (N1, N2, N3, N4), object placement time (N1, N2, N3, N4), maximum grasp aperture time (N1, N2, N3, N4), and wrist path length (N1, N2, N3, N4). Greenhouse-Geisser corrections were applied when sphericity assumptions were violated. Significant main effects were followed by Bonferroni-corrected pairwise comparisons, controlling for experiment-wise error at α = 0.05. Subsequently, to test whether increases in movement duration could be explained solely by increased spatial displacement (i.e., wrist path), we performed Spearman correlation analyses between wrist path length and each of the four segmented temporal components: Movement Initiation Time, Time of Maximal Grasp Aperture, Reaching Time, and Object Placement Time. Spearman's method was chosen. Full results are reported and visualized in Supplementary Figure S1.

## Results

### Total movement time

**Figure 7:**
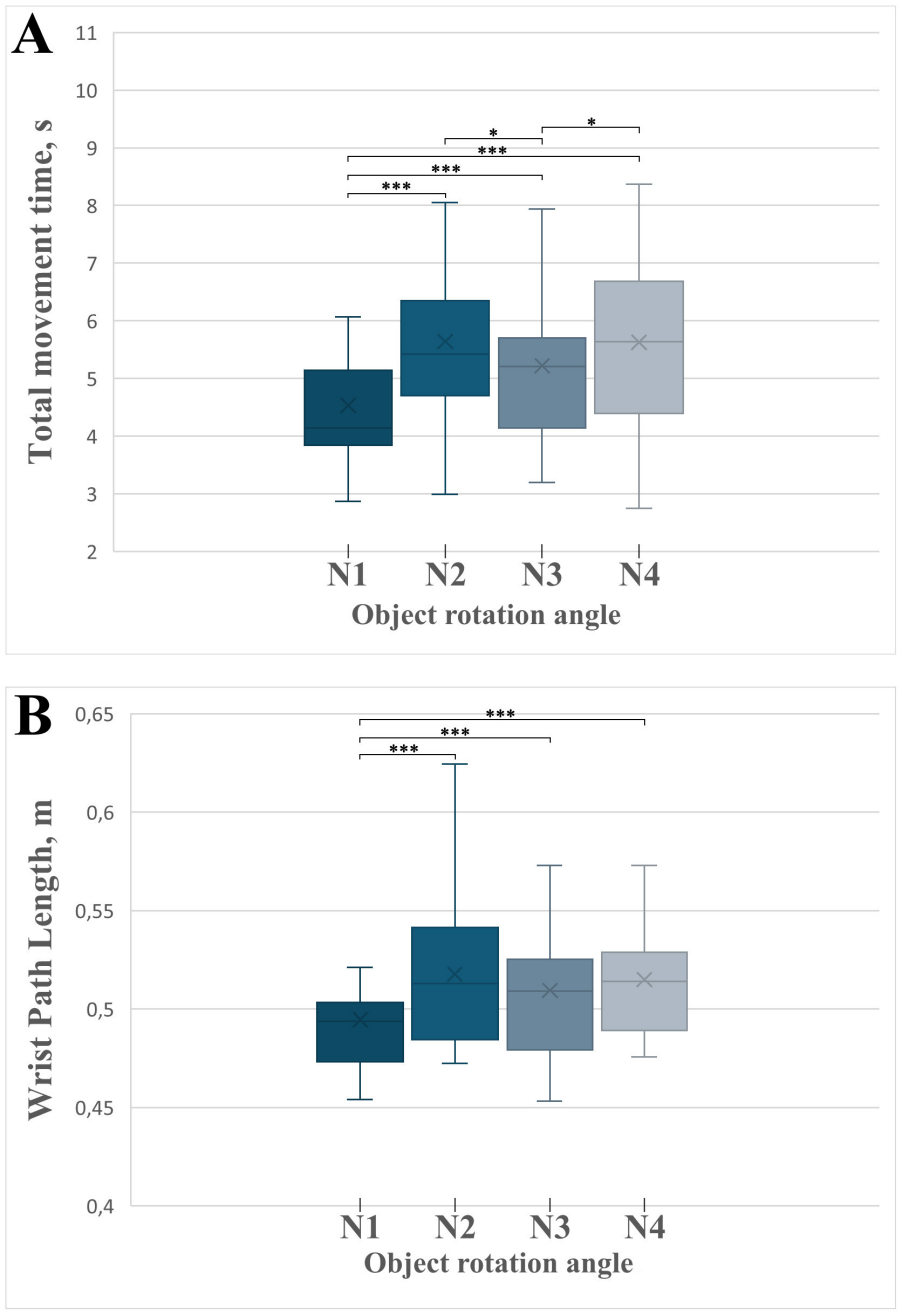
Boxplots illustrating the temporal parameters of the complete grasp-and-place movement phases across different experimental conditions defined by object rotation angles (N1 = 0°, N2 = 90°, N3 = 180°, N4 = 270°). Asterisks indicate statistically significant differences between conditions: *p < 0.05, ***p < 0.001. Error bars represent standard deviation. **(A)** Total movement time across rotation angle conditions. **(B)** Wrist path length across rotation angle conditions.

We observed a significant effect of the object rotation angle on the total movement time [*F*_(3)_ = 25.232, MSE = 5.664, *p* < 0.001, partial η^2^ = 0.558; Figure 7A]. *Post-hoc* comparisons (Supplementary Table S1) revealed that total movement time was significantly shorter in the non-rotated condition (0° rotation, N1) compared to all rotated conditions: 90° (N2), 180° (N3), and 270° (N4; all *p* < 0.001). These findings clearly indicate that movements performed without rotation, where object and platform orientations are congruent, are executed more rapidly. Additionally, movements involving a 180° rotation (N3) were significantly faster than those involving 90° (N2; *p* = 0.011) and 270° rotations (N4; *p* = 0.022), highlighting that symmetrical rotations facilitate quicker movements compared to asymmetrical rotations.

### Movement initiation time

**Figure 8:**
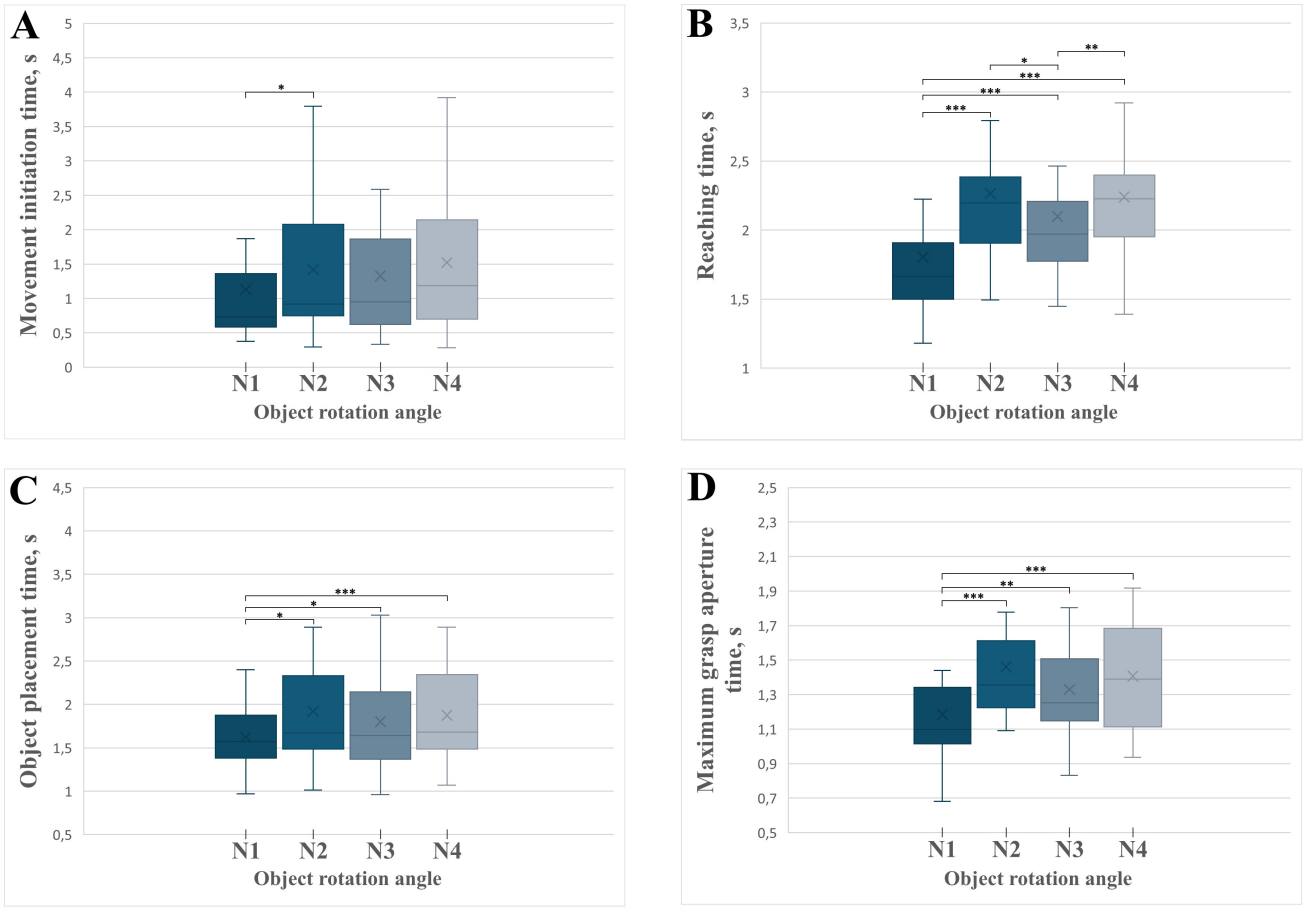
Boxplots illustrating the temporal parameters of distinct grasp-and-place movement phases across experimental conditions with varying object rotation angles (N1 = 0°, N2 = 90°, N3 = 180°, N4 = 270°). Significant differences between conditions are marked with asterisks: *p < 0.05, **p < 0.01, ***p < 0.001. Error bars indicate standard deviations. **(A)** Movement initiation time across different rotation angles. **(B)** Reaching time required to grasp the object. **(C)** Object placement time across rotation conditions. **(D)** Time of maximum grasp aperture across rotation conditions.

The movement initiation time varied significantly across rotation conditions [*F*_(3)_ = 6.244, MSE = 0.587, *p* < 0.001, partial η^2^ = 0.238; Figure 8A]. *Post-hoc* tests (Supplementary Table S1) revealed that movements without rotation (N1) began significantly faster than those requiring a 90° rotation (N2; *p* = 0.038). No significant differences emerged between other rotation angles, suggesting that the complexity of initial decision-making and motor planning is particularly sensitive to the introduction of rotation itself, especially when transitioning from no rotation to initial rotational complexity (90°).

### Reaching time

There was a significant effect of the object rotation angle on the time required to reach and grasp the object [*F*_(3)_ = 41.657, MSE = 0.938.006, *p* < 0.001, partial η^2^ = 0.676; Figure 8B]. *Post-hoc* analyses (Supplementary Table S1) revealed significantly shorter reaching times in the non-rotated condition (N1) compared to all rotated conditions (90°, 180°, 270°; all *p* < 0.001). Moreover, the reaching times for the 180° rotation condition (N3) were significantly shorter than those for the 90° rotation condition (N2; *p* = 0.026) and the 270° rotation condition (N4; *p* = 0.002). These results suggest that symmetrical rotations (180°) enable faster and presumably more efficient reaching movements compared to more asymmetric rotations.

### Object placement time

The object placement time, defined as the interval from object grasp to placement, significantly varied across rotation conditions [*F*_(3)_ = 7.899, MSE = 0.368, *p* < 0.001, partial η^2^ = 0.283; Figure 8C]. *Post-hoc* comparisons (Supplementary Table S1) indicated significantly shorter object movement times in the non-rotated condition (N1) compared to all rotation conditions: 90° (N2, *p* = 0.019), 180° (N3, *p* = 0.023), and 270° (N4, *p* < 0.001). No significant differences were found among rotation angles (N2, N3, N4), indicating that the critical determinant of movement duration was the requirement of rotation itself rather than the magnitude of rotation.

### Time of maximal grasp aperture

The rotation angle significantly affected the time required to reach the maximal grasp aperture [*F*_(3)_ = 11.152, MSE = 0.301, *p* < 0.001, partial η^2^ = 0.358; Figure 8D]. *Post-hoc* tests (Supplementary Table S1) revealed significantly shorter times to maximal aperture in the non-rotated condition (N1) compared to all rotated conditions: 90° (*p* < 0.001), 180° (*p* = 0.002), and 270° (*p* < 0.001). The differences among the rotation conditions (N2, N3, and N4) were not significant. This outcome suggests increased complexity and time demands for grasp preparation whenever rotation is necessary, irrespective of rotation magnitude.

### Wrist path length

The wrist trajectory length during reaching was significantly influenced by the object rotation angle [*F*_(3)_ = 17.127, MSE = 0.002, *p* < 0.001, partial η^2^ = 0.461; Figure 7B]. *Post-hoc* analyses (Supplementary Table S1) confirmed significantly shorter wrist paths in the non-rotated condition (N1) compared to all rotation-required conditions (90°, 180°, 270°; all *p* < 0.001). These results clearly indicate that anticipated object rotation increases reaching trajectory complexity, likely due to preparatory adjustments in hand positioning aimed at facilitating subsequent rotation tasks.

### Correlation between path length and temporal segments

Spearman correlations between wrist path length and segmented movement times revealed no significant association with Movement Initiation Time (ρ = 0.0191, *p* = 0.48), indicating that increased movement distance does not account for preparatory planning latency. Time of Maximal Grasp Aperture showed a weak but significant positive correlation with wrist path length (ρ = 0.2827, *p* < 0.001), suggesting some anticipatory shaping effects on trajectory. A moderate and statistically robust correlation was found for Reaching Time (ρ = 0.4375, *p* < 0.001), indicating that spatial demands partially influence reach phase duration. Object Placement Time did not significantly correlate with wrist path (ρ = −0.0184, *p* = 0.50). These results highlight that not all components of movement duration scale with spatial extent, particularly the initiation phase, which reflects anticipatory control.

## Discussion

In this study, we established and validated a structured methodological framework to investigate the impact of object rotation on motor planning during reach-to-grasp movements. By employing precise temporal segmentation based on positional tracking data, we successfully delineated the distinct phases of grasp-related movements, specifically isolating movement initiation, the time to reach the maximum grasp aperture, the reaching phase, and the object placement time. This approach is consistent with standard methodologies in motor control research, emphasizing the importance of clearly separating motor preparation from execution (Jeannerod, 1997; Wong et al., 2015; Castiello, 2005; Haith et al., 2016; van Vliet et al., 2013). The critical insight provided by our paradigm is the explicit demonstration that motor planning constitutes a distinct phase that is intricately linked with subsequent execution parameters.

Our analysis of total movement time revealed significant increases when rotations of the object were needed, emphasizing that object manipulation involving rotation introduces heightened cognitive and motor demands compared with direct placement without rotation (Milivojevic et al., 2011; Jovanovic and Schwarzer, 2017; Jost and Jansen, 2022). Interestingly, movements requiring a 180° rotation were executed faster than those involving 90° and 270° rotations. This unexpected finding suggests that not all rotational adjustments impose identical cognitive and motor costs. The facilitation observed for the 180° rotation condition could be explained by biomechanical symmetry, potentially aligning more naturally with habitual hand configurations and thus simplifying motor planning processes (Milivojevic et al., 2011; Jovanovic and Schwarzer, 2017; Jost and Jansen, 2022; Smeets and Brenner, 1999). These results challenge the simplistic view that increased rotation angles always correspond directly to increased complexity, highlighting that the relationship between motor planning and execution efficiency is nuanced and dependent not only on rotation magnitude but also on the biomechanical and perceptual properties of movements. Future studies should systematically explore the influence of object symmetry and perceptual familiarity on rotational grasping strategies.

Considering movement initiation time, our data revealed significant delays, specifically when transitioning from no rotation to a 90° rotation condition. These delays likely reflect increased decision-making and preparatory demands at intermediate rotational complexities, underscoring that even small increments in rotation can substantially influence motor preparation (Glover, 2004). The absence of significant differences across other rotation conditions could indicate a threshold effect, whereby initial cognitive demands become elevated as soon as rotation is introduced, plateauing thereafter. Nevertheless, given the limited sample size in our study, these interpretations remain preliminary. A larger participant cohort would be necessary to substantiate these findings and clarify whether rotation complexity uniformly influences motor planning at the initiation phase or if specific angles disproportionately affect preparation strategies.

Analysis of the reaching phase demonstrated that future motor requirements, i.e., object rotations occurring after grasp completion, profoundly influenced earlier movement stages. This finding corroborates earlier studies highlighting the anticipatory nature of motor planning, where subsequent movement complexity modifies initial motor execution strategies (Castiello, 2005; Paulignan et al., 1997). Movements associated with a 180° rotation again demonstrated superior efficiency compared with those involving 270°, reinforcing the hypothesis that perceptual and biomechanical alignment facilitates more efficient motor planning. This anticipatory adaptation suggests that the motor system proactively integrates the anticipated spatial demands of the entire movement sequence, adjusting initial trajectories accordingly. Such predictive control highlights the integrated nature of motor planning, where execution is continuously modulated on the basis of upcoming motor demands rather than being confined to discrete segments.

Our observations regarding object movement time reinforce the idea that the primary determinant of execution efficiency is the requirement of rotation itself rather than rotation magnitude. Although intuitively, larger rotations might appear mechanically more demanding and thus more time-consuming, our data indicate that cognitive and motor planning components predominantly dictate object placement efficiency (Herbort et al., 2014). This underscores the fundamental role of cognitive preparation and anticipatory motor planning in shaping movement execution outcomes. Future studies employing neurophysiological methods (e.g., EEG, TMS) could provide further insights into how specific brain regions associated with action preparation (e.g., the premotor cortex) modulate these cognitive and motor demands during rotational tasks.

The analysis of the maximal grasp aperture time further highlighted the complexity introduced by the anticipated rotations, as the aperture timings significantly differed between the rotation-required and non-rotation conditions. The requirement to subsequently rotate an object likely compels early adjustments in hand configuration, increasing preparatory complexity (Santello et al., 2002). Notably, our analysis of positional tracking data revealed previously unreported multiple finger aperture cycles, initial finger release from the starting position followed by grasp-specific aperture adjustment. This finding emphasizes the need to refine current models of grasp preparation, moving beyond traditional single-cycle assumptions. Future research should investigate whether these dual cycles represent a generalized motor strategy or reflect specific experimental setup constraints, potentially informing rehabilitation strategies for grasping impairments in clinical populations (Wing et al., 1986). Notably, our detailed analysis revealed a dual-cycle structure in the thumb–index finger distance profile: an initial release from the starting position, followed by grasp-specific preshaping. This previously unreported phenomenon may reflect a transition between two distinct motor sub-goals, postural disengagement and object-directed motor planning. While this behavior has not been extensively characterized in the literature, it conceptually aligns with prior work on multi-stage grasp planning (Wing et al., 1986; Castiello et al., 1998), which emphasizes the adaptability of finger movement strategies under different preparatory conditions. Future studies may explore whether this dual-cycle structure is a generalized motor feature or task-specific. While our use of non-sensical geometric objects was designed to minimize semantic bias and standardize task complexity, it necessarily reduces ecological validity. This choice allowed us to isolate motor planning and execution processes without the influence of learned object functions or affordances. However, we acknowledge that the behaviors observed may differ from naturalistic grasping actions performed with everyday items. Future studies could extend this work by integrating familiar or functional objects to examine how contextual and semantic information shapes anticipatory motor control in more realistic scenarios. Moreover, wrist trajectory path analysis provided compelling evidence of anticipatory motor adjustments. Specifically, significantly longer wrist paths in conditions requiring rotation suggest proactive positional adjustments of the wrist and hand even before the object grasps. This aligns with evidence from other studies demonstrating that motor planning extends beyond simple reaction measures, entailing sophisticated, continuous online adjustments influenced by anticipated motor demands (Hoff and Arbib, 1993). Additionally, our correlation analysis between wrist path length and segmented movement times provides further support for the dissociation between anticipatory motor planning and movement execution. Notably, Movement Initiation Time, which is our operational proxy for motor planning, was not significantly associated with movement distance, reinforcing its cognitive origin rather than biomechanical dependence. In contrast, Reaching Time showed a moderate correlation with wrist path length, suggesting that once planning is complete, trajectory length contributes substantially to reaching duration. The weak correlation with Time of Maximal Grasp Aperture may reflect anticipatory shaping adjustments, although its modest strength limits interpretation. These findings bolster our methodological framework by empirically demonstrating that motor planning cannot be reduced to simple distance-based metrics. This adds strong value for future applications of the protocol in clinical populations where planning and execution deficits may be differentially affected. We also examined whether participants exhibited general performance drift across the 64 trials, which could reflect learning or fatigue effects. A quartile-based analysis, collapsed across all rotation conditions, revealed modest improvements in total movement time and object placement time from the first to the last quartile, consistent with common learning trends in motor behavior. However, this effect was assessed independently of the rotation manipulation. Crucially, the main findings reported in this study, namely, the significant effects of object rotation on movement initiation, reaching time, and grasp aperture, were derived from separate condition-specific analyses and remain statistically robust. Thus, even if modest learning occurred, the randomization of conditions across the session mitigates the risk of systematic bias. The observed learning trend does not confound our core interpretation of anticipatory motor planning. Full results of the performance drift analysis are presented in Supplementary Table S2 and Supplementary Figure S2.

Our results reinforce the concept that motor control mechanisms are inherently predictive, continuously integrating future action requirements into early-stage motor execution.

## Conclusions

Our methodological paradigm effectively isolates motor planning from execution phases, enabling a detailed investigation into how anticipated object rotations influence reach-to-grasp movements. The clear segmentation approach allows for rigorous examination of the cognitive and motor demands associated with varying rotation angles, highlighting both anticipatory and reactive motor control strategies. Our findings provide strong evidence that motor planning is deeply integrated with motor execution, continuously adapting to future movement demands.

Despite its strengths, our study has several limitations. While our methodology robustly separates motor phases, future studies should integrate complementary neurophysiological techniques (e.g., TMS, EEG) to directly investigate the underlying neural mechanisms associated with motor planning. Employing such multimodal approaches could deepen our understanding of how cortical networks, particularly premotor and parietal regions, mediate anticipatory control strategies in complex motor tasks. Moreover, the generalizability of our findings is limited by the relatively homogeneous and healthy young adult sample. Future studies should replicate and extend this work in more diverse populations, including older adults and individuals with neurological impairments, to assess the broader applicability of the proposed paradigm.

Additionally, the use of manually operated occlusion glasses introduces a potential source of temporal variability across trials. Although we mitigated this by defining trial onset objectively, using the maximum distance between two reflective markers attached to the glasses, future studies should consider implementing electronically controlled occlusion systems (e.g., electrochromic lenses) to further enhance temporal precision and experimental standardization. Furthermore, although our sample included both male and female participants, it was not designed or powered to evaluate sex-based differences in motor planning or execution. As a result, potential effects of inter-individual variability, including sex, were not formally assessed. Future research should consider stratifying by this factor to better understand how individual traits influence anticipatory motor control.

In addition to its value for fundamental motor control research, the current paradigm may have translational applications in the assessment of motor deficits in patients with neurological conditions such as stroke (Osumi et al., 2019) or Parkinson's disease (Fasano et al., 2022; Vissani et al., 2021). The controlled and reproducible nature of the task enables broader applicability across research and clinical domains. By clearly isolating cognitive-motor planning components from motor execution, our paradigm offers potential diagnostic and rehabilitative advantages. Future investigations should extend this approach to clinical populations (e.g., stroke patients and Parkinson's disease patients), exploring whether specific impairments in motor planning phases predict overall functional outcomes or respond differently to targeted interventions.

## Data Availability

The original contributions presented in the study are included in the article/Supplementary material, further inquiries can be directed to the corresponding author.
