# Supplementary material for: A comprehensive approach to studying motor planning and execution using 3D-printed objects and motion tracking technology
Source: Front Hum Neurosci. 2025 Jun 25;19:1620526. doi: 10.3389/fnhum.2025.1620526 (PMC12238092; doi:10.3389/fnhum.2025.1620526)
Supplement: Supplementary file 2 [file Table_1.pdf]

Supplementary Table S1

Table 1. Results of post hoc pairwise comparisons for total movement time across rotation conditions.

Group	Comparison group	Mean difference	Std. error	p	95 % confidence interval for the variance	
					Lower bound	Upper bound
N1	N2	-1.109	0.164	<0.001	-1.588	-0.630
	N3	-0.688	0.109	<0.001	-1.008	-0.368
	N4	-1.092	0.169	<0.001	-1.586	-0.598
N2	N1	1.109	0.164	<0.001	0.630	1.588
	N3	0.421	0.117	0.011	0.078	0.764
	N4	0.017	0.179	1.000	-0.508	0.541
N3	N1	0.688	0.109	<0.001	0.368	1.008
	N2	-0.421	0.117	0.011	-0.764	-0.078
	N4	-0.404	0.123	0.022	-0.765	-0.044
N4	N1	1.092	0.169	<0.001	0.598	1.586
	N2	-0.017	0.179	1.000	-0.541	0.508
	N3	0.404	0.123	0.022	0.044	0.765

Table 2. Results of post hoc pairwise comparisons for movement initiation time across rotation conditions.

Group	Comparison group	Mean difference	Std. error	p	95 % confidence interval for the variance	
					Lower bound	Upper bound
N1	N2	-0.290	0.095	0.038	-0.567	-0.012
	N3	-0.198	0.071	0.070	-0.408	0.011
	N4	-0.394	0.141	0.067	-0.806	0.019
N2	N1	0.290	0.095	0.038	0.012	0.567
	N3	0.091	0.062	0.938	-0.090	0.272
	N4	-0.104	0.092	1.000	-0.372	0.164
N3	N1	0.198	0.071	0.070	-0.011	0.408
	N2	-0.091	0.062	0.938	-0.272	0.090
	N4	-0.195	0.087	0.218	-0.450	0.060
N4	N1	0.394	0.141	0.067	-0.019	0.806
	N2	0.104	0.092	1.000	-0.164	0.372
	N3	0.195	0.087	0.218	-0.060	0.450

Table 3. Results of post hoc pairwise comparisons for reaching time across rotation conditions.

Group	Comparison group	Mean difference	Std. error	p	95 % confidence interval for the variance	
					Lower bound	Upper bound
N1	N2	-0.460	0.059	<0.001	-0.633	-0.287
	N3	-0.294	0.028	<0.001	-0.376	-0.212
	N4	-0.436	0.042	<0.001	-0.558	-0.313
N2	N1	0.460	0.059	<0.001	0.287	0.633
	N3	0.166	0.051	0.026	0.015	0.316
	N4	0.024	0.057	1.000	-0.141	0.190
N3	N1	0.294	0.028	<0.001	0.212	0.376
	N2	-0.166	0.051	0.026	-0.316	-0.015
	N4	-0.142	0.032	0.0015	-0.235	-0.048
N4	N1	0.436	0.042	<0.001	0.313	0.558
	N2	-0.024	0.057	1.000	-0.190	0.141
	N3	0.142	0.032	0.0015	0.048	0.235

Table 4. Results of post hoc pairwise comparisons for object placement time across rotation conditions.

Group	Comparison group	Mean difference	Std. error	p	95 % confidence interval for the variance	
					Lower bound	Upper bound
N1	N2	-0.301	0.090	0.019	-0.564	-0.038
	N3	-0.180	0.055	0.023	-0.342	-0.019
	N4	-0.255	0.047	<0.001	-0.394	-0.116
N2	N1	0.301	0.090	0.019	0.038	0.564
	N3	0.120	0.051	0.171	-0.029	0.269
	N4	0.045	0.086	1.000	-0.207	0.298
N3	N1	0.180	0.055	0.023	0.019	0.342
	N2	-0.120	0.051	0.171	-0.269	0.029
	N4	-0.075	0.056	1.000	-0.240	0.090
N4	N1	0.255	0.047	<0.001	0.116	0.394
	N2	-0.045	0.086	1.000	-0.298	0.207
	N3	0.075	0.056	1.000	-0.090	0.240

Table 5. Results of post hoc pairwise comparisons for time to maximum grasp aperture across rotation conditions.

Group	Comparison group	Mean difference	Std. error	p	95 % confidence interval for the variance	
					Lower bound	Upper bound
N1	N2	-0.275	0.048	<0.001	-0.416	-0.135
	N3	-0.145	0.033	0.0018	-0.243	-0.047
	N4	-0.222	0.038	<0.001	-0.333	-0.110
N2	N1	0.275	0.048	<0.001	0.135	0.416
	N3	0.130	0.069	0.435	-0.071	0.331
	N4	0.054	0.068	1.000	-0.146	0.253
N3	N1	0.145	0.033	0.0018	0.047	0.243
	N2	-0.130	0.069	0.435	-0.331	0.071
	N4	-0.077	0.034	0.226	-0.178	0.024
N4	N1	0.222	0.038	<0.001	0.110	0.333
	N2	-0.054	0.068	1.000	-0.253	0.146
	N3	0.077	0.034	0.226	-0.024	0.178

Table 6. Results of post hoc pairwise comparisons for wrist path length during reaching across rotation conditions.

Group	Comparison group	Mean difference	Std. error	p	95 % confidence interval for the variance	
					Lower bound	Upper bound
N1	N2	-0.023	0.005	<0.001	-0.037	-0.010
	N3	-0.015	0.003	<0.001	-0.023	-0.006
	N4	-0.020	0.002	<0.001	-0.026	-0.014
N2	N1	0.023	0.005	<0.001	0.010	0.037
	N3	0.008	0.004	0.388	-0.004	0.021
	N4	0.003	0.004	1.000	-0.009	0.015
N3	N1	0.015	0.003	<0.001	0.006	0.023
	N2	-0.008	0.004	0.388	-0.021	0.004
	N4	-0.005	0.003	0.287	-0.013	0.002
N4	N1	0.020	0.002	<0.001	0.014	0.026
	N2	-0.003	0.004	1.000	-0.015	0.009
	N3	0.005	0.003	0.287	-0.002	0.013
