# Supplementary material for: A comprehensive approach to studying motor planning and execution using 3D-printed objects and motion tracking technology
Source: Front Hum Neurosci. 2025 Jun 25;19:1620526. doi: 10.3389/fnhum.2025.1620526 (PMC12238092; doi:10.3389/fnhum.2025.1620526)
Supplement: Supplementary file 3 [file Table_2.pdf]

Supplementary Table S2

Table 1. Results of post hoc pairwise comparisons for total movement time across quartiles.

Group	Comparison group	Mean difference	Std. error	p	95 % confidence interval for the variance	
					Lower bound	Upper bound
Q1	Q2	0.399	0.128	0.012	0.060	0.738
	Q3	0.304	0.146	0.225	-0.082	0.690
	Q4	0.714	0.127	0.000	0.377	1.052
Q2	Q1	-0.399	0.128	0.012	-0.738	-0.060
	Q3	-0.095	0.157	1.000	-0.511	0.320
	Q4	0.315	0.132	0.107	-0.036	0.666
Q3	Q1	-0.304	0.146	0.225	-0.690	0.082
	Q2	0.095	0.157	1.000	-0.320	0.511
	Q4	0.410	0.157	0.057	-0.008	0.828
Q4	Q1	-0.714	0.127	0.000	-1.052	-0.377
	Q2	-0.315	0.132	0.107	-0.666	0.036
	Q3	-0.410	0.157	0.057	-0.828	0.008

Table 2. Results of post hoc pairwise comparisons for movement initiation time across quartiles.

Group	Comparison group	Mean difference	Std. error	p	95 % confidence interval for the variance	
					Lower bound	Upper bound
Q1	Q2	0.296	0.074	0.000	0.100	0.492
	Q3	0.115	0.107	1.000	-0.168	0.399
	Q4	0.320	0.075	0.000	0.120	0.520
Q2	Q1	-0.296	0.074	0.000	-0.492	-0.100
	Q3	-0.180	0.111	0.636	-0.475	0.115
	Q4	0.024	0.064	1.000	-0.144	0.193
Q3	Q1	-0.115	0.107	1.000	-0.399	0.168
	Q2	0.180	0.111	0.636	-0.115	0.475
	Q4	0.205	0.109	0.369	-0.085	0.494
Q4	Q1	-0.320	0.075	0.000	-0.520	-0.120
	Q2	-0.024	0.064	1.000	-0.193	0.144
	Q3	-0.205	0.109	0.369	-0.494	0.085

Table 3. Results of post hoc pairwise comparisons for reaching time across rotation quartiles.

Group	Comparison group	Mean difference	Std. error	p	95 % confidence interval for the variance	
					Lower bound	Upper bound
Q1	Q2	0.117	0.060	0.315	-0.042	0.276
	Q3	0.227	0.052	0.000	0.088	0.365
	Q4	0.364	0.050	0.000	0.230	0.498
Q2	Q1	-0.117	0.060	0.315	-0.276	0.042
	Q3	0.110	0.050	0.175	-0.023	0.243
	Q4	0.248	0.049	0.000	0.118	0.377
Q3	Q1	-0.227	0.052	0.000	-0.365	-0.088
	Q2	-0.110	0.050	0.175	-0.243	0.023
	Q4	0.138	0.042	0.008	0.025	0.250
Q4	Q1	-0.364	0.050	0.000	-0.498	-0.230
	Q2	-0.248	0.049	0.000	-0.377	-0.118
	Q3	-0.138	0.042	0.008	-0.250	-0.025

Table 4. Results of post hoc pairwise comparisons for object placement time across quartiles.

Group	Comparison group	Mean difference	Std. error	p	95 % confidence interval for the variance	
					Lower bound	Upper bound
Q1	Q2	-0.013	0.062	1.000	-0.177	0.152
	Q3	-0.038	0.079	1.000	-0.247	0.172
	Q4	0.030	0.069	1.000	-0.154	0.215
Q2	Q1	0.013	0.062	1.000	-0.152	0.177
	Q3	-0.025	0.089	1.000	-0.262	0.212
	Q4	0.043	0.089	1.000	-0.192	0.279
Q3	Q1	0.038	0.079	1.000	-0.172	0.247
	Q2	0.025	0.089	1.000	-0.212	0.262
	Q4	0.068	0.097	1.000	-0.188	0.324
Q4	Q1	-0.030	0.069	1.000	-0.215	0.154
	Q2	-0.043	0.089	1.000	-0.279	0.192
	Q3	-0.068	0.097	1.000	-0.324	0.188

Table 5. Results of post hoc pairwise comparisons for time to maximum grasp aperture across quartiles.

Group	Comparison group	Mean difference	Std. error	p	95 % confidence interval for the variance	
					Lower bound	Upper bound
Q1	Q2	0.089	0.047	0.361	-0.036	0.214
	Q3	0.165	0.040	0.000	0.057	0.272
	Q4	0.218	0.043	0.000	0.105	0.332
Q2	Q1	-0.089	0.047	0.361	-0.214	0.036
	Q3	0.076	0.039	0.338	-0.029	0.180
	Q4	0.129	0.041	0.009	0.022	0.237
Q3	Q1	-0.165	0.040	0.000	-0.272	-0.057
	Q2	-0.076	0.039	0.338	-0.180	0.029
	Q4	0.054	0.035	0.763	-0.039	0.147
Q4	Q1	-0.218	0.043	0.000	-0.332	-0.105
	Q2	-0.129	0.041	0.009	-0.237	-0.022
	Q3	-0.054	0.035	0.763	-0.147	0.039

Table 6. Results of post hoc pairwise comparisons for wrist path length during reaching across quartiles.

Group	Comparison group	Mean difference	Std. error	p	95 % confidence interval for the variance	
					Lower bound	Upper bound
Q1	Q2	0.089	0.047	0.361	-0.036	0.214
	Q3	0.165	0.040	0.000	0.057	0.272
	Q4	0.218	0.043	0.000	0.105	0.332
Q2	Q1	-0.089	0.047	0.361	-0.214	0.036
	Q3	0.076	0.039	0.338	-0.029	0.180
	Q4	0.129	0.041	0.009	0.022	0.237
Q3	Q1	-0.165	0.040	0.000	-0.272	-0.057
	Q2	-0.076	0.039	0.338	-0.180	0.029
	Q4	0.054	0.035	0.763	-0.039	0.147
Q4	Q1	-0.218	0.043	0.000	-0.332	-0.105
	Q2	-0.129	0.041	0.009	-0.237	-0.022
	Q3	-0.054	0.035	0.763	-0.147	0.039
