# Supplementary figures and images for: A comprehensive approach to studying motor planning and execution using 3D-printed objects and motion tracking technology
Source: Front Hum Neurosci. 2025 Jun 25;19:1620526. doi: 10.3389/fnhum.2025.1620526 (PMC12238092; doi:10.3389/fnhum.2025.1620526)

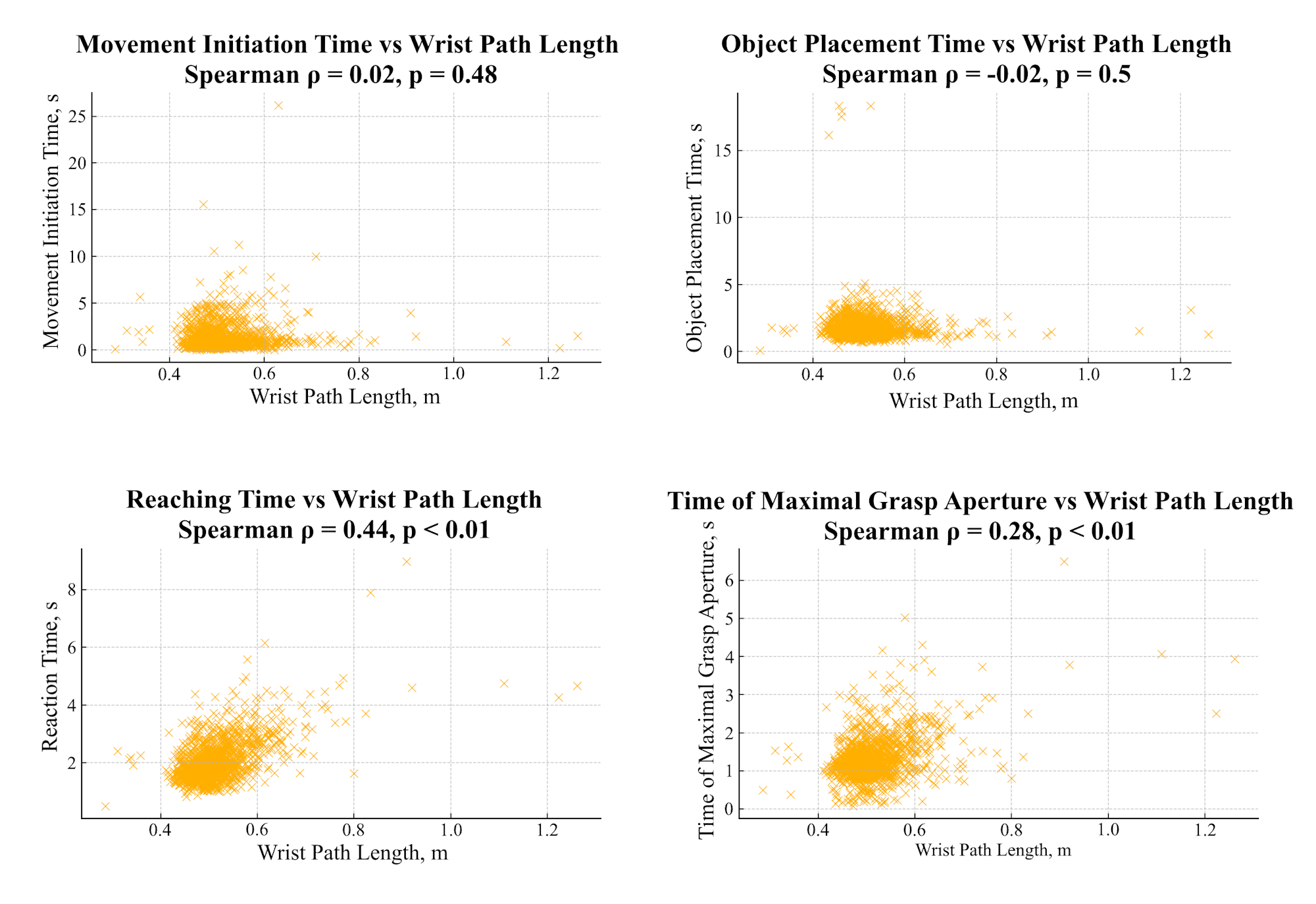

Supplement: Supplementary file 4 [file Image_1.tiff]

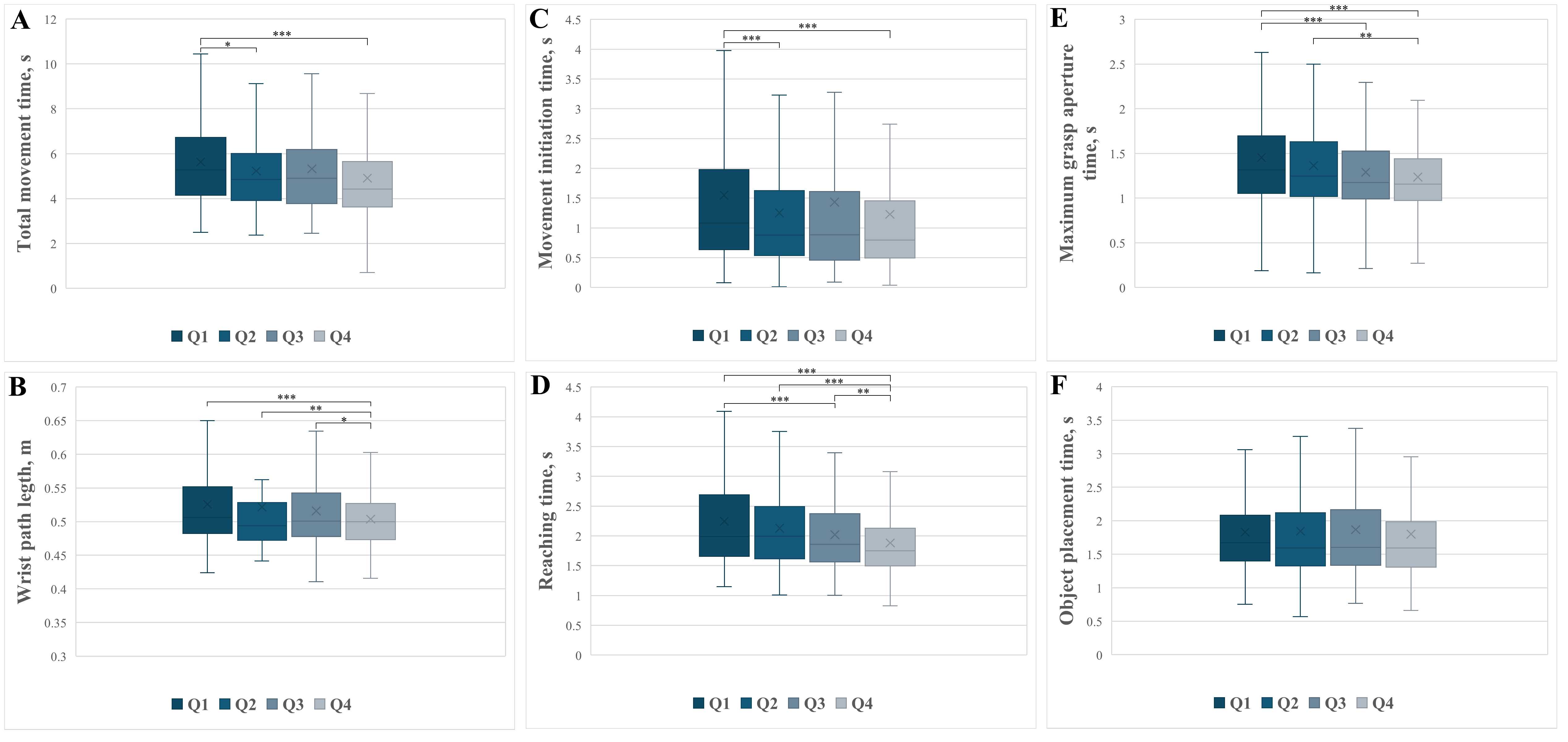

Supplement: Supplementary file 5 [file Image_2.tiff]
